# A Bibliometric Analysis of P2X7R in Cardiovascular Diseases from 2005 to 2024

**DOI:** 10.2174/011573403X376830250619055153

**Published:** 2025-06-25

**Authors:** Juanjuan Tan, Wenqi Zhou, Zhiye Guo, Li Cheng, Yalong Kang, Qiong Wang, Jing Dong, Haifang Wang, Qi Zhang, Li Shen, Kai Huang

**Affiliations:** 1 Institute of Integrative Medicine, Shaanxi University of Chinese Medicine, Xianyang, 712046, China;; 2 Shaanxi Key Laboratory of Integrated Traditional and Western Medicine for Prevention and Treatment of Cardiovascular Diseases, Shaanxi University of Chinese Medicine, Xianyang, 712083, China;; 3 The Second Clinical Medical College, Shaanxi University of Chinese Medicine, Xianyang, 712083, China;; 4 Institute of Mechanobiology & Medical Engineering, School of Life Sciences & Biotechnology, Shanghai Jiao Tong University, 800 Dongchuan Road, Minhang, 200240, Shanghai, China;; 5 Department of Cardiology, The Second Affiliated Hospital of Shaanxi University of Traditional Chinese Medicine, Xianyang, China;; 6 Shaanxi University of Chinese Medicine Affiliated Hospital, Xianyang, 712000, China;; 7 Shaanxi Key Laboratory of Chinese Medicine Encephalopathy, Shaanxi University of Chinese Medicine, Xianyang, 712046, China;; 8 Cardiology Department, The Third Hospital of Changsha, Changsha, 413000, China;; 9 Key Laboratory for the Genetics of Developmental and Neuropsychiatric Disorders, Ministry of Education, Shanghai Jiao Tong University, 800 Dongchuan Road, Minhang, 200240, Shanghai, China

**Keywords:** P2X7R, cardiovascular disease, bibliometric analysis, CiteSpace, VOSviewer, purinergic signaling

## Abstract

**Introduction:**

The P2X7 receptor (P2X7R), which mediates inflammation, is implicated in an extensive variety of diseases, including cardiovascular dysfunction. Recently, studies focusing on the role of P2X7R in cardiovascular disorders have garnered significant attention. However, a bibliometric evaluation within this area has yet to be carried out.

**Methods:**

A bibliometric analysis was performed by searching for research related to P2X7R and cardiovascular diseases in the Web of Science Core Collection (WoSCC) database from 2005 to 2024. The tools CiteSpace and VOSviewer were utilized to analyze data and create visual representations of various elements, including countries, institutions, authors, journals and keywords.

**Results:**

Over the past two decades, 371 articles in English were obtained in the last 20 years. The People's Republic of China, Nanchang University, the journal 'Purinergic Signalling,' and author Shandong Liang had the highest productivity in their respective categories. The top 4 keywords were “activation',' “p2x7 receptor',' “ATP',' and “inflammation”. Burst keyword analysis indicated that “purinergic signaling” and “oxidative stress” are emerging key areas worthy of further investigation. These topics, seeing a surge in interest, are predicted to remain prominent in research.

**Discussion:**

This is the first bibliometric analysis of P2X7R in cardiovascular disorders, which reports the hot spots and emerging trends. The interaction between “purinergic signaling”, “inflammation”, and “oxidative stress” are considered to be the current research priorities, suggesting that these topics are likely to remain central in future research.

**Conclusion:**

This study underscores the growing importance of P2X7R in cardiovascular research and offers valuable insights to guide future investigations.

## 1 INTRODUCTION

Cardiovascular Diseases (CVDs), a primary cause of mortality globally, encompass conditions such as Acute Myocardial Infarction (AMI), heart failure, Atrial Fibrillation (AF), atherosclerosis, aortic aneurysm, vein graft failure, hypertension and diabetic vascular complications [1-12]. Clinical treatment for CVDs primarily focuses on etiology-directed pharmacotherapy, including antihypertensive agents, statins, and antidiabetic medications [13]. However, existing drug treatments often fail to achieve satisfactory results. Therefore, it is urgent to explore new mechanisms regarding the occurrence and development of cardiovascular diseases to help develop new therapeutic drugs and strategies. The P2X7 receptor (P2X7R) has emerged as a key regulator of inflammatory and oxidative pathways in CVDs; however, a systematic analysis of its research landscape are lacking. While prior narrative reviews summarized the biological mechanisms of P2X7R in CVDs [14] and systematic reviews evaluated its therapeutic potential [15], critical knowledge gaps persist in understanding the evolution of this field, such as temporal trends, collaboration patterns, and translation gaps. Firstly, no study has quantified whether P2X7R research is accelerating in specific CVD subtypes. Secondly, the dominance of certain countries/institutions may influence research priorities; however, this has not been systematically mapped. Additionally, disconnects between basic research hotspots and clinical trial topics remain unmeasured. Therefore, we performed the first bibliometric study to map P2X7R research trends in CVDs.

Purinergic receptors are a class of cell membrane receptors sensitive to purine compounds that play a crucial role in intercellular signaling and involve multiple physiological processes, such as neurotransmission, platelet aggregation, cardiac function, immune responses, glucose-dependent insulin release, and cellular growth [16-19]. Purinergic receptors are classified into two main types: P1 and P2 receptors [20]. The P2 receptor is further divided into P2X receptors (ion channel types) and P2Y receptors (G protein-coupled types). In mammals, seven P2X receptor subtypes (P2X1 to P2X7) have been cloned. Recent studies have found that P2X7 receptors are closely associated with the development of cardiovascular diseases through their involvement in inflammatory responses, promotion of neurotransmitter release and induction of apoptosis, making them important targets for drug development and therapeutic research [21]. Besides, P2X7, a nonselective cation channel, is used as a potential new anti-inflammatory medicine [22, 23] and drug delivery system for hydrophilic substances in photodynamic therapy [24]. Notably, plant natural products are found as a source of new P2 receptor ligands, highlighting the pharmacological potential of natural compounds targeting P2 receptors [25]. Therefore, exploring the evolution and cutting-edge trends in this research area holds profound importance. However, no bibliometric examination has been performed on this specific topic.

Bibliometrics is a burgeoning method of knowledge synthesis that assesses both the quantitative and qualitative characteristics of publications and investigates dominant research trends within specific research fields [26]. With the swift growth of scientific research, the importance of bibliometric analysis of scholarly publications has significantly increased [27]. Recently, computational and visual analytic technologies, such as CiteSpace and VOSviewer, have offered sophisticated approaches for conducting bibliometric analysis within specialized fields [28]. Therefore, this research employed two commonly utilized bibliometric software; CiteSpace and VOSviewer, leveraging data from the Web of Science Core Collection (WoSCC) encompassing publications from 2005 to 2024. This research represents the inaugural attempt to delineate the present landscape and focal points concerning P2X7R in cardiovascular diseases over the past two decades. This study aims to achieve three objectives: (1) to identify the most productive authors, institutions and countries/regions contributing significantly to the field through their articles and publications, (2) to delineate the core research topics and areas currently attracting interest and (3) to forecast the future trajectories of P2X7R research in the context of cardiovascular diseases.

## 2 BIBLIOMETRIC METHODS

### 2.1 Data Collection

The data used in this study was systematically searched from the WoSCC. The search query was configured as TS = (P2X purinergic receptor 7 or P2X7R or P2X7R or P2X7 Purinoceptor or P2X purinoceptor 7) and TS = (coronary disease or myocardia heart disease or ischemic event or peripheral atrial or aortic disorder or aortic aneurysm or ventricular impairment or stroke or intracerebral hemorrhage or cerebrovascular accident or hypertension). The parameters were established to include all articles published from September 30, 2005, to September 30, 2024. English research articles were filtered along with comments that were not retracted for further analysis. A total of 371 articles were retrieved, exporting all records and cited articles as either tab-delimited or plain text files. These exported files were labeled as “download_*.txt” and earmarked for subsequent analysis. Bibliometric analysis of P2X7R for cardiovascular diseases in the workflow is shown in the Graphical abstract.

### 2.2 Data Analysis

In this research, a combination of bibliometric and visual analysis techniques were employed using tools like Microsoft Excel 2019, CiteSpace 6.3. R1 (64 bit) and VOSviewer 1.6.20. The analysis of annual publication frequency, citation counts, contributing institutions, geographical distribution, and other pertinent data was accomplished using Microsoft Excel. For the interpretation and visual representation of large-scale data sets in an easily comprehensible format, VOSviewer was was utilized. This tool facilitated the visualization of various aspects, such as country/region distribution, institutional contributions, author collaborations, and keyword co-occurrence journal coupling maps. To pinpoint rapidly evolving topic keywords and burgeoning areas of study, a keyword burst detection analysis was performed using CiteSpace. For this research, the parameters were configured as follows: a time slice ranging from 2005 to 2024, with each slice representing a year, and a g-index k set at 25.

## 3 RESULTS

### 3.1 Basic Analysis of the Literature

Fig. (**1**) depicts the trend in the volume of the 371 articles included in the study. There was a general upward trend in the number of publications from 2005 to 2024, with a rapid increase observed from 2005 to 2015. However, the period from 2015 to 2024 observed a fluctuating trend in the annual output of articles. It was noteworthy that the year 2024 showed a slight decline, which may be due to the fact that the current analysis was conducted only up to September 2024, resulting in incomplete data.

### 3.2 Countries/Regions Analysis

The research on the treatment of cardiovascular diseases with P2X7R was globally distributed, with contributions from 46 countries or regions. Fig. (**2A**) presented the top ten contributors based on the number of publications. China led the field with 132 papers, followed by the USA with 84 and England with 39. The interaction and collaboration among these countries were represented by seven distinct clusters in Figs. (**2B**-**D**). A significant increase in the number of publications from China was evident from 2020 onwards, as shown in Fig. (**2C**).

### 3.3 Institutions Analysis

**Fig. (3):**
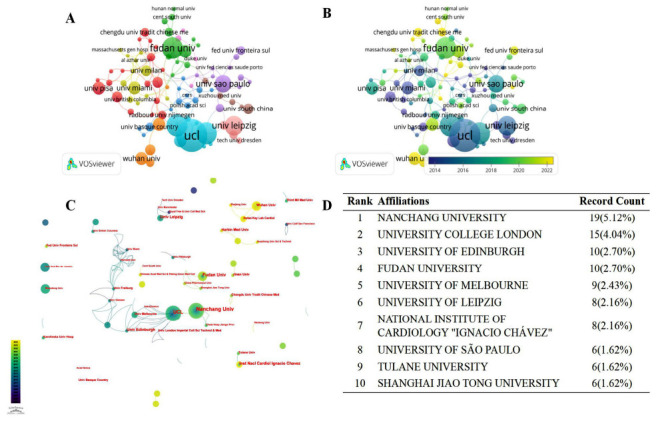
Institutions relationship and collaboration density and the top 10 institutions. **A**. Network visualization of various research institutions. **B**. Overlay visualization of various research institutions. **C**. Analysis of institutional cooperation based on CiteSpace visual map. **D**. The top 10 institutions.

Fig. (**3**) reveals the participation of a total of 579 universities and institutes in the study. Figs. (**3A**-**D**) illustrate that Nanchang University leads with the highest number of publications, contributing 19 articles. Following closely are University College London with 15 articles and both the University of Edinburgh and Fudan University, with 10 publications each. The University of Melbourne contributed 9 articles, securing its place among the top contributors. The top 10 productivity institutions are universities or research institutes, and three are from China (Fig. **3D**). These results show that China is at the forefront of the world. In terms of timeline, the University College London showed a higher publication output in 2015, while Fudan University saw an increase in publications starting from 2020 onward.

### 3.4 Authors’ Analysis

The study of P2X7R in cardiovascular diseases involved contributions from a total of 2234 authors. Among these, Shangdong Liang, with 16 publications, Guilin Li, with 14 publications, and Burnstock Geoffrey, with 11 publications, emerged as the most prolific contributor. The co-authorship network is represented in Figs. (**4A** and **B**), where authors are color-coded into different clusters to signify their closely related groups. In the green cluster, Liang S., Li G., and Liu S. were all affiliated with the Medical College, Nanchang University. They collaboratively found that P2X7R played a role in the pathophysiological process of myocardial ischemic injury [29, 30] and its gene polymorphism was associated with primary hypertension in postmenopausal women [31].

### 3.5 Journals Analysis

The study incorporated 371 publications that were distributed across 191 different journals. Table **1** lists the top 10 journals, among which Purinergic Signalling stands out with the maximum number of publications, totaling 31 articles. This is followed by the International Journal of Molecular Sciences with 15 articles and Frontiers in Physiology with 9. The top three journals constitute 58.51% of the total publications in the top 10 journals. The average impact factor (IF) of these leading 10 journals is 4.35.

### 3.6 Journals Co-citation and Coupling Analysis

**Fig. (5):**
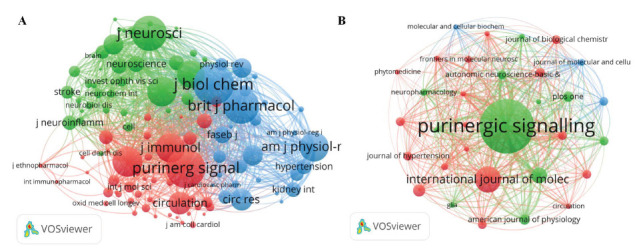
Journal co-citation and journal coupling. **A**. Journal co-citation analysis; **B**. Journal coupling analysis.

In the journal co-citation analysis, VOSviewer extracted 2831 articles, from which 112 papers were selected with a citation frequency greater than 50 for presentation (Fig. **5A**). Based on the total citation intensity analysis, the top-ranked journals are “Journal of Biological Chemistry”, “Purinerg Signal”, and “British Journal of Pharmacology”. For the journal coupling analysis, a minimum coupling amount of three was set and 32 out of 191 journals met this criterion (Fig. **5B**). According to the coupling strength, the top three journals are “Purinergic Signaling”, “International Journal of Molecular Sciences”, and “Frontiers in Physiology”.

### 3.7 Literature Theme and Keyword Analysis

Reviewing the literature reveals the focal areas and subjects of research interest. We conducted a series of cluster analyses and traced the chronological development of literature that was frequently co-cited. For the analysis of keywords, VOSviewer was employed to generate a visual map of keyword interconnections, as depicted in Fig. (**6**). The magnitude of the circles corresponds to the overall connectivity, while the width of the lines signifies the frequency of co-occurrences. As demonstrated in Fig. (**6**) and Table **2**, 76 keywords have shown up more than 10 times in the analyzed publications. The most common keywords are “Activation”, “P2X7R receptor”, “Expression”, “ATP”, and “Extracellular ATP”. These keywords highlight the central themes and concepts in the body of research on P2X7R in cardiovascular diseases. In Fig. (**6**), the red cluster is mainly composed of “activation”, “P2X7 receptor”, “inflammation”, and “NOD-Like Receptor Protein 3 (NLRP3) inflammasome”. This cluster focuses on the diverse and important roles that P2X7R plays in inflammatory responses. Under pathological conditions, such as tissue hypoxia or inflammation, the release and accumulation of large amounts of ATP activate P2X7R, leading to non-selective cation channel opening, allowing ions such as K^+^, Na^+^ and Ca^2+^ to pass through, further triggering inflammatory responses [32]. The blue clusters were mainly composed of “P2X7R receptor”, “ATP”, and “Extracellular ATP”. ATP serves as the natural ligand for P2X7R, with a marked increase under conditions of tissue hypoxia or inflammation. The binding of ATP to the P2X7R can activate the NLRP3 inflammasome, thereby exacerbating the inflammatory response [33]. The green cluster is represented by “oxidative stress”, “apoptosis”, and “cardiovascular disease”. Oxidative stress can lead to cell apoptosis and endothelial dysfunction and subsequently trigger cardiovascular diseases [34].

### 3.8 Keywords with the Strongest Citation Bursts

The surge in certain keywords indicates the focal points and emerging patterns of interest within the domain. Fig. (**7**) illustrates a list of keywords that have exhibited the most significant citation surge, arranged in chronological sequence. The burst strength denotes the degree of fluctuation in the frequency of a term over a specific timeframe [35]. The keyword “ATP” experienced a notable citation surge in 2007, highlighting its significance in early research. “ATP release” also experienced a burst in 2008, indicating interest in its release mechanisms, while “extracellular ATP” in the same year suggested its role in purinergic signaling.

In 2017, the “NLRP3 inflammasome” gained attention, reflecting its relevance in inflammation linked to various diseases, including cardiovascular conditions. The “P2X7 receptor”, crucial for ATP signaling, maintained interest from 2005 to 2018, peaking in 2017. Keywords such as “intracerebral hemorrhage” and “stroke” also surged in 2017, emphasizing the role of purinergic signaling in cerebrovascular diseases. “Myocardial infarction” showed sustained interest from 2007 to 2024, peaking in 2020. “Purinergic signaling” experienced a resurgence in 2021, supported by “oxidative stress”, which peaked in 2015. “Heart failure” also experienced a burst in 2017, indicating ongoing research into purinergic signaling in heart diseases. Overall, the data reflects a growing focus on purinergic signaling in cardiovascular conditions, highlighting its potential for therapeutic intervention.

## 4 DISCUSSION

### 4.1 General Information

The P2X7R, as a member of the purinergic P2X receptor family, is primarily expressed in endothelial cells, cardiac smooth muscle cells, and immune cells [21]. Its endogenous ligand ATP is significantly released under conditions of tissue hypoxia or inflammation [36]. Upon binding with ATP, the P2X7R can activate the NLRP3 inflammasome, thereby exacerbating the inflammatory response [37]. Recent studies have revealed the pathological roles of the P2X7R in cardiovascular diseases, such as atherosclerosis, hypertension, myocardial infarction, and pulmonary arterial hypertension [21]. This article aims to explore the research progress of the P2X7R in cardiovascular diseases using bibliometric analysis.

This study employed bibliometric methods to examine the evolution and key trends of P2X7R research in cardiovascular diseases over the past two decades. The analysis revealed that China led in terms of publication volume (132 papers), followed by the USA (84 papers) and England (39 papers). China's leading position in this field may be due to the following reasons. Firstly, China's substantial investment in research and development. Over the past few decades, China has significantly increased its R&D expenditure, both in absolute terms and as a percentage of its Gross Domestic Product (GDP). This financial commitment has undoubtedly played a major role in propelling the country to the forefront of numerous scientific disciplines, including the field of visualization analysis. Secondly, the sheer size of China's population provides a vast talent pool for scientific research. With a population exceeding 1.4 billion, even a small percentage of individuals engaged in research translates to a large number of researchers. This demographic advantage, coupled with a robust educational system that emphasizes science and technology, contributes to a high output of scientific research. Lastly, the role of government policies in fostering scientific growth in China should not be underestimated. The Chinese government has implemented various initiatives aimed at promoting scientific and technological progress. These range from policies designed to attract overseas-educated researchers to significant funding programs for research projects and the establishment of research institutes and innovation hubs. These combined factors have created an environment conducive to scientific research and innovation, which is reflected in China's leading position in the field of visualization analysis. However, it is also important to note that the dynamics of scientific research are complex and multifaceted, and these factors are part of a larger system that contributes to China's scientific output. Further study is needed to fully understand the interplay of these and other factors in shaping the landscape of scientific research in China and around the world.

Notably, Nanchang University and University College London have emerged as significant regional research hubs. Among the leading contributors, Shangdong Liang from Nanchang University was the most productive author. The research was disseminated across 191 different journals, with Purinergic Signalling, International Journal of Molecular Sciences, and Frontiers in Physiology being the most frequently featured. Half of the top 10 journals were categorized under Medicine, while Neuroscience journals represented 20% of the total. This suggests that research on P2X7R in cardiovascular diseases predominantly appears in journals specializing in medicine and neuroscience.

The author with the most published articles in this field is Shangdong Liang. He and his research team members (Guilin Li, Shuangmei Liu, and Yun Gao) have been extensively involved in researching the role of P2X receptors in neuropathic pain and the modulation of sensory nerves related to cardiovascular diseases [38, 39]. The research discovered that the P2X7R is instrumental in a range of physiological and pathological events, with a special emphasis on modulating pain and transmitting sensory information [38, 40]. Activation of the P2X7R has been linked to chronic pain conditions, and its antagonists show promise in alleviating pain symptoms. Additionally, the purinergic signaling system—involving the P2X7R—emerges as a potential therapeutic target for conditions like depression and chronic pain. In the context of pain modulation, the P2X7R is prominently involved in sensory transmission and regulation, notably within the Dorsal Root Ganglion (DRG). Studies suggest that regulating the expression of the P2X7R in the DRG, such as through NONRATT021972 siRNA intervention, can modulate neuropathic pain behaviors in diabetic rats [29]. Moreover, in the cardiovascular context, the P2X7R's impact extends to regulating neural activities associated with cardiovascular diseases. Manipulating P2X7R expression in the superior cervical ganglion through interventions like NONRATT021972 siRNA can modulate sympathetic nervous system activity, offering novel avenues for cardiovascular disease treatment. Additionally, substances like gallic acid have shown efficacy in alleviating chronic pain and depressive symptoms in rodents, achieved by suppressing the ferroptosis processes mediated by the P2X7R [41]. However, gallic acid effectively mitigates neuropathic pain behavior in rats by inhibiting the P2X7R-mediated NF-κB/STAT3 signaling pathway [42]. Upregulation of P2X7R in the Superior Cervical Ganglia (SCG) following myocardial ischemia and its contribution to enhanced sensory-sympathetic coupling, as well as excitatory effects on the sympathetic nerves, highlight its critical role in cardiovascular pathophysiology. Inhibition of P2X7R in the stellate ganglion could prevent the exacerbation of sensory-sympathetic coupling induced by myocardial ischemic injury, indicating the receptor's importance in the crosstalk between sensory and sympathetic neurons [39]. Targeting P2X7R in the stellate ganglion may mitigate this enhanced coupling and reduce sympathetic reflexes, potentially offering therapeutic benefits in cardiovascular pathologies. Therefore, the multifaceted role of the P2X7R in pain modulation, sensory transmission, and cardiovascular interactions underscores its significance as a potential therapeutic target in various pathological conditions, providing new insights and avenues for future research and treatment strategies [43].

Geoffrey Burnstock is a prominent author whose scholarly works are frequently referenced in academic circles. Burnstock proposed the significant concept of a receptor family known as purinergic receptors in 1978. These can be further divided into two subclasses: P1 and P2 [44]. The authors mentioned above are esteemed in academia due to their extensive research contributions in elucidating the mechanisms of purinergic receptors, the prevention and treatment of neuropathic pain and the modulation of sensory nerves related to cardiovascular diseases.

### 4.2 Hotspots and Frontiers

Through keyword analysis, we pinpointed some of the most crucial research focuses concerning P2X7R in cardiovascular diseases over the past two decades. These include: 1) The interaction between ATP, its release and P2X7R, 2) NLRP3 inflammasome and 3) oxidative stress.

ATP serves as a fundamental energy currency in cells as well as functions as an important signaling molecule in various physiological processes [45]. ATP release from cells, particularly through purinergic receptors such as P2X receptors, plays a crucial role in intercellular communication and modulating cellular responses [46]. Among the P2X receptor family, P2X7R has garnered significant attention due to its involvement in diverse cellular functions and pathophysiological conditions [47]. Research focusing on the interplay between ATP, ATP release mechanisms, and P2XR7 has unveiled several key areas of interest in the scientific community. One prominent area is the role of ATP and P2XR7 in neurobiology [48, 49]. Studies have demonstrated the involvement of ATP-P2XR7 signaling in synaptic transmission, neuronal excitability, and neuroinflammation, highlighting the importance of purinergic signaling in neural function and dysfunction [48, 50, 51]. Furthermore, investigations into the immune system have revealed the impact of ATP release and P2XR7 activation on inflammatory responses [32, 52, 53]. ATP acts as a danger signal, triggering the activation of immune cells through P2XR7, leading to the release of pro-inflammatory cytokines and the formation of inflammasomes. Understanding the intricate crosstalk between ATP, P2XR7, and immune cells is crucial for deciphering the mechanisms underlying inflammatory diseases and exploring potential therapeutic targets. In the context of cancer biology, the ATP-P2XR7 axis has emerged as a critical player in the tumor microenvironment [54]. ATP released from cancer cells acts as a signaling molecule, influencing immune responses, tumor growth, and metastasis. P2XR7 activation in tumor cells has been linked to various processes, such as cell proliferation, angiogenesis, and resistance to chemotherapy, underscoring its significance as a potential target for cancer therapy [55, 56]. Moreover, the investigation of ATP release mechanisms, including vesicular release, connexin hemichannels, and pannexin channels, has provided insights into the diverse ways by which cells release ATP into the extracellular space [57]. These mechanisms not only regulate purinergic signaling but also contribute to the modulation of cellular functions and intercellular communication in various physiological and pathological contexts. In brief, the dynamic interplay between ATP, ATP release mechanisms, and P2XR7 represents a rich area of research with significant implications for neurobiology, immunology, cancer biology, and beyond. Unraveling the complexities of these interactions holds promise for advancing our understanding of cellular signaling, disease mechanisms, and the development of novel therapeutic strategies targeting ATP-P2XR7 signaling pathways. Continued exploration of these research avenues is essential for harnessing the full potential of ATP and P2XR7 in both physiological and pathological conditions.

NLRP3 and P2X7R have emerged as key players in the pathophysiology of cardiovascular diseases, attracting significant research attention due to their intricate roles in inflammation, cell death and vascular dysfunction [37, 58]. The NLRP3 inflammasome, a multiprotein complex involved in innate immunity and inflammatory responses, has been implicated in the development and progression of cardiovascular disorders, such as myocardial infarction, atherosclerosis and stroke [59, 60]. The abnormal activation of the NLRP3 inflammasome is associated with a variety of diseases, encompassing diabetes, metabolic syndrome, atherosclerosis, as well as cardiovascular and neurodegenerative disorders [61]. While, the activation of the P2X7R triggers a series of downstream effects, such as oxidative stress, inflammatory reactions, and cell death [62, 63]. Recent advances in the field have highlighted the potential of targeting NLRP3 and P2X7R as therapeutic strategies for cardiovascular diseases [15, 21, 64, 65]. Pharmacological inhibitors of NLRP3 or P2X7R have shown promise in preclinical studies by attenuating atherosclerotic thrombosis, heart failure, and recurrent pericarditis [59, 66]. Furthermore, genetic manipulation of NLRP3 or P2X7R expression in animal models has provided valuable insights into their roles in vascular pathology and disease outcomes, paving the way for the development of targeted interventions to mitigate cardiovascular disorders [67, 68]. Future research directions may involve elucidating the specific molecular mechanisms by which NLRP3 and P2X7R contribute to vascular inflammation, atherosclerosis, and thrombosis, exploring their interactions with other signaling pathways, and evaluating the efficacy of novel therapeutic approaches targeting these molecules in clinical settings. Understanding the complex roles of NLRP3 and P2X7R in cardiovascular diseases is crucial for developing precision medicine strategies to improve patient outcomes and reduce the burden of these debilitating conditions.

Oxidative stress, characterized by an imbalance between the production of Reactive Oxygen Species (ROS) and antioxidant defenses, is recognized as a significant factor in the pathogenesis of endothelial dysfunctions and CVDs [2, 69-76]. ROS, while essential for normal physiological functions, can induce detrimental effects when produced in excess, thereby contributing to the progression of CVDs. Recent investigations have unveiled the intricate role of oxidative stress in the inflammatory response and endothelial dysfunction, which are the two key pathophysiological processes in CVDs [77, 78]. Oxidative stress triggers inflammatory cascades leading to the activation of pro-inflammatory cytokines and adhesion molecules, which subsequently promote leukocyte adhesion and infiltration, a hallmark of early atherosclerotic changes [79]. Concurrently, oxidative stress-induced endothelial dysfunction, primarily mediated by reduced nitric oxide availability, contributes to impaired vasodilation, pro-thrombotic state, and vascular inflammation [80]. Moreover, oxidative stress has been shown to modulate cellular signaling pathways that underpin the development and progression of atherosclerosis and heart failure. ROS can activate matrix metalloproteinases, leading to the degradation of the extracellular matrix, a critical step in plaque destabilization and rupture in atherosclerosis [81]. In the context of heart failure, oxidative stress can induce cardiomyocyte apoptosis and fibrosis through the activation of various signaling pathways, including the MAPK and NF-kB pathways [82, 83].

Purinergic signaling—extracellular signaling mediated by purine nucleotides and nucleosides—plays important roles in many diseases [56, 84-90]. Research has shown that abnormal purinergic signaling contributes to the pathogenesis of various cardiovascular disorders, such as atherosclerosis [91-96], hypertension [90, 97-103], and ischemia [104-106]. Specifically, the P2X7R, as a subtype of the P2 purinergic receptors, is primarily expressed in immune cells, cardiac smooth muscle cells, and endothelial cells. Its natural ligand, ATP, is significantly released under conditions of tissue hypoxia or inflammation. Upon binding with ATP, the P2X7R activates the NLRP3-dependent inflammasome, thereby exacerbating the inflammatory response. Recent research has unveiled the pathological role of the P2X7R in cardiovascular diseases, such as atherosclerosis, hypertension, myocardial infarction and pulmonary arterial hypertension [107-110]. P2X7R plays a significant role in the onset and progression of hypertension. Previous studies have shown elevated levels of P2X7R in rat models under hypertensive conditions [111]. Research by Zhao *et al.* discovered that plasma ATP concentrations significantly increase in hypertensive patients [112]. High concentrations of ATP activate the expression of P2X7R in Antigen-Presenting Cells (APCs), subsequently mediating the upregulation of CD86 and inducing inflammatory responses that damage the body. A study by Xu *et al.* revealed that the level of P2X7R is associated with salt-sensitive hypertension and kidney damage [113]. The use of a P2X7R blocker (A-438079) in animal models significantly reduced blood pressure, slowed renal interstitial fibrosis, improved creatinine clearance and mitigated inflammatory cell infiltration. Another study indicated that a specific P2X7R inhibitor, Brilliant Blue G (BBG), effectively restored renal medulla blood flow, reduced proteinuria, improved local inflammation and delayed the progression of hypertensive kidney damage [108]. In addition, the single nucleotide polymorphism (rs591874) of the P2X7R is associated with changes in systolic and diastolic blood pressure, particularly in relation to nocturnal diastolic pressure, suggesting that the P2X7R plays a role in maintaining the steady state of hypertension [114]. The above studies suggest that the expression of the P2X7R increases in hypertension. The use of P2X7R inhibitors not only lowers blood pressure but also delays the onset of hypertension-related complications. This suggests that the P2X7R may become an important clinical target for treating hypertension, offering a new direction for future hypertension treatment research.

Under pathological conditions (such as tissue hypoxia or inflammation), the release and accumulation of large amounts of ATP activate the P2X7R, leading to the opening of non-selective cation channels, which facilitate the passage of ions like K^+^, Na^+^, Ca^2+^, and further trigger inflammatory responses [115]. P2X7R is closely associated with the activation of the NLRP3 inflammasome. When Duewell *et al.* transplanted bone marrow lacking NLRP3 or Asc synthesis into mice deficient in low-density lipoprotein receptors, they observed a significant reduction in early atherosclerosis and inflammasome-dependent IL-18 levels [116]. Moreover, when Stachon *et al.* used a P2X7R-deficient mouse model, they found that the deficiency of the P2X7R could reduce the occurrence and development of atherosclerosis, and they also observed an increased expression of the P2X7R in human atherosclerotic plaques [117]. These findings suggest that the P2X7R and the inflammatory responses it mediates play a crucial role in the development of atherosclerosis and indicate that inhibiting the P2X7R may be a potential direction for its treatment. In acute myocardial infarction, the P2X7R may play a crucial role through various mechanisms. Firstly, the activation of the P2X7R promotes inflammatory responses, endothelial dysfunction and atherosclerosis, all of which are key factors in the development of myocardial infarction [117, 118]. Studies have shown that in atherosclerotic mouse models, the expression of the P2X7R significantly increases in macrophages within lesion areas, leading to the excessive phagocytosis of ox-LDL through monocytes and macrophages, accelerating the formation of foam cells and subendothelial plaques and thus promoting atherosclerosis. In contrast, mice lacking the P2X7R have smaller atherosclerotic lesions after a high-cholesterol diet [117]. Secondly, inhibiting the activation of the P2X7R can reduce the activity of the AMPK/MAPK signaling pathway and decrease the expression of the Extracellular Matrix Metalloproteinase Inducer (EMMPRIN) and MMP9. EMMPRIN and MMP9 are related to late-stage atherosclerotic lesions and their increase can lead to plaque rupture and myocardial infarction [119]. Furthermore, an increased expression of the P2X7R is associated with an increased number of cell deaths in ischemia-reperfusion injury. The use of small interfering RNA (siRNA) targeting the P2X7R or inhibitors can prevent inflammasome assembly and reduce the area of myocardial infarction in mouse models [109]. Research suggests that P2X7R plays a potentially significant role in the development of pulmonary arterial hypertension, primarily through its involvement in the proliferation and phenotypic transformation of PASMCs under hypoxic conditions *via* the JNK signaling pathway. Under hypoxic conditions, the expression of the P2X7R in PASMCs is significantly higher than at normal oxygen levels. The use of the P2X7R inhibitor significantly alleviates the pathological changes in lung tissue caused by hypoxia. Further research has found that treatment with a P2X7R agonist exacerbates the proliferation and synthetic phenotype of PASMCs under hypoxia. Conversely, the use of siRNA targeting the P2X7R or inhibitors significantly reduces the hypoxia-induced upregulation of phosphorylated JNK/JNK in PASMCs [120]. Therefore, targeting purinergic signaling and specifically the P2X7R, opens up new therapeutic possibilities for these disorders. Potential strategies could include the development of drugs that block the P2X7R or modulate its activity, thereby reducing inflammation.

Several P2X7R inhibitors have been investigated in preclinical and clinical studies for a variety of conditions, largely due to the receptor's role in inflammation. These include compounds like JNJ-47965567, A804598 and AK1780, among others. JNJ-47965567 is a centrally permeable, high-affinity P2X7 antagonist that exhibited modest yet significant efficacy in the rat model of neuropathic pain [121]. JNJ-47965567 can attenuate chemically-induced kindling in seizure models [122] and could ameliorate Amyotrophic Lateral Sclerosis (ALS) progression in SOD1G93A mice [123]. A804598, which is a novel, potent and selective P2RX7 antagonist, contributes to cytotoxicity through the suppression of glycolysis and AKT activation in human HCC [124, 125]. A804598 also inhibits inflammation in the brain and liver in C57BL/6J mice exposed to chronic ethanol and high-fat diet [126]. Besides, the latest research found that AK1780, a novel P2X7R inhibitor, inhibited calcium influx in human or rat P2X7R-expressing cells. More interestingly, the blockade of P2X7R with AK1780 in the CNS can have analgesic properties without any CNS adverse effects [127]. However, clinical trial results have been inconsistent. As of our last update, a P2X3R antagonist, Gefapixant (also known as MK-7264, RO 4926219, AF-219), was in Phase 3 trials for the treatment of refractory chronic cough and demonstrated promising results [128]. As of September 2024, no clinical trials have been reported in terms of cardiovascular diseases and pulmonary hypertension. However, given the growing body of preclinical evidence suggesting a role for P2X7R in these conditions, it is possible that such trials could be conducted in the future. By integrating these translational research findings with bibliometric trends, we can gain a better understanding of the potential clinical applications of P2X7R inhibitors and the future direction of the field. However, it is important to note that translating preclinical findings to successful clinical applications can be a complex and challenging process, and not all promising preclinical results lead to effective treatments in humans.

## 5 LIMITATIONS

Several possible constraints exist in this study. Initially, the bibliometric data solely originated from WoSCC. Non-English literatures were excluded. Secondly, the search methodology might not encompass all research within this domain. Additionally, publication categories like corrections and retractions could impact data analysis outcomes. Finally, the rapid development of the field may cause the analysis to overlook recent research advances. While this study focuses on descriptive trend analysis, future work could incorporate multi-dimensional metrics and employ machine-learning approaches to refine trend predictions.

The bibliometric analysis identifies oxidative stress, inflammation, and purinergic signaling as key words in P2X7R research does provide valuable insights, but translating these findings into actionable clinical insights requires further exploration and validation. Bibliometric analysis primarily focuses on identifying research trends and patterns through data mining and statistical analysis. While it highlights the importance of oxidative stress, inflammation, and purinergic signaling in P2X7R research, these findings are more reflective of research focus areas rather than direct clinical applications. The analysis may show a high frequency of studies on P2X7R-mediated inflammation, but it does not inherently provide specific clinical interventions or therapeutic targets. Clinical insights often require data from patient cohorts, clinical trials, or translational studies. Bibliometric analysis, however, is based on published literature and does not directly involve patient data. As a result, it may fail to capture the nuances and complexities of clinical practice, such as patient variability, comorbidities and real-world treatment outcomes.

## CONCLUSION

To our knowledge, this research represents the first extensive bibliometric analysis of P2X7R in cardiovascular diseases spanning from 2005 to 2024. Utilizing tools like CiteSpace and VOSviewer, we discerned the knowledge distribution traits of P2X7R in cardiovascular diseases. “Oxidative stress”, “apoptosis”, “ATP”, and “inflammation” emerged as key themes in this area. P2X7R plays a crucial role in cardiovascular diseases, and its increased expression may exacerbate inflammatory responses, leading to greater harm to the body. Therefore, P2X7R inhibitors show promise in alleviating the impact of these diseases and are anticipated to emerge as a hot topic in upcoming studies. In conclusion, this research offers valuable insights for consolidating advancements in the field and delving into prospective research avenues.

## Figures and Tables

**Fig. (1):**
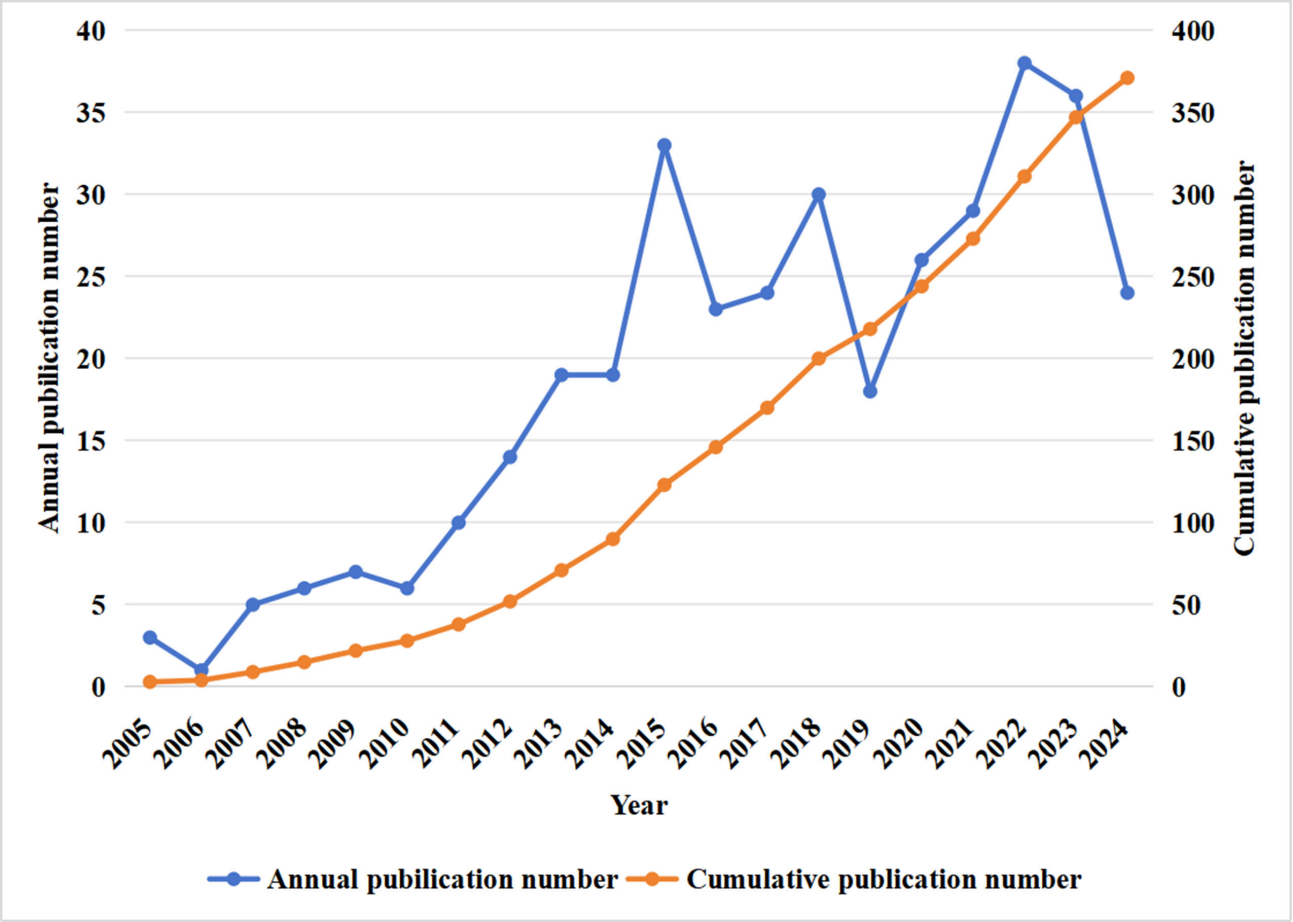
Annual number of publications and cumulative number of publications from 2005 to 2024.

**Fig. (2):**
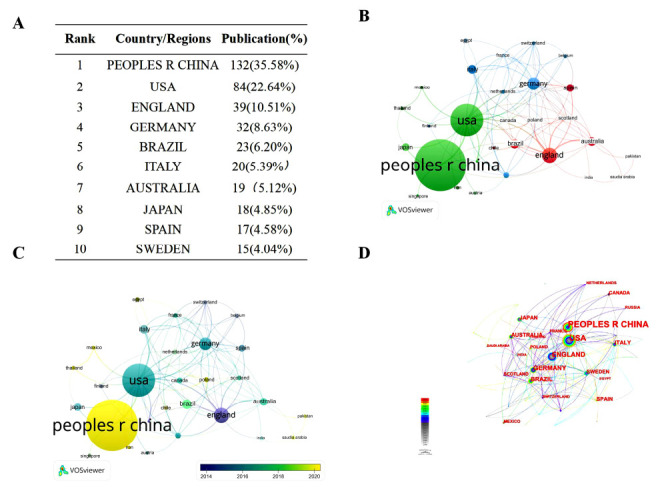
Link density relationships between countries/regions and the top ten countries/regions. **A**. The top ten countries/regions. The People's Republic of China (n=132) published the most papers, followed by the United States (n=84) and the United Kingdom (n=39); **B**. Network visualization of countries/ regions associations; **C**. Overlay visualization of countries/ regions associations; **D**. The intensity of cooperation between countries/ regions.

**Fig. (4):**
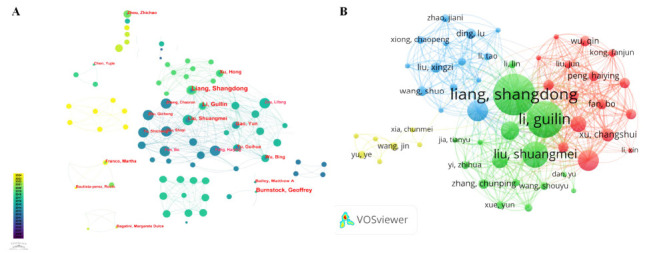
Author collaboration network. **A**. Author co-authorship network analysis based on CiteSpace visual map; **B**. Author co-authorship network analysis based on VOSviewer visual map.

**Fig. (6):**
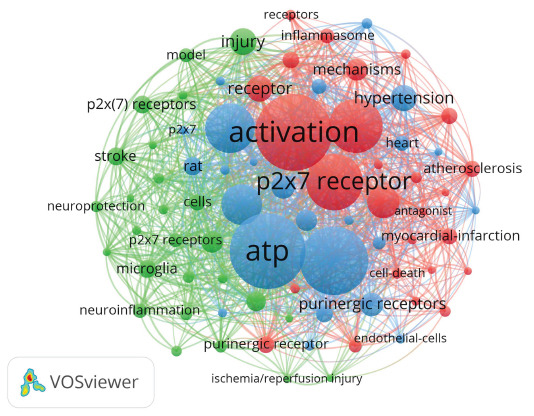
Cluster analysis and cooperative network of keywords.

**Fig. (7):**
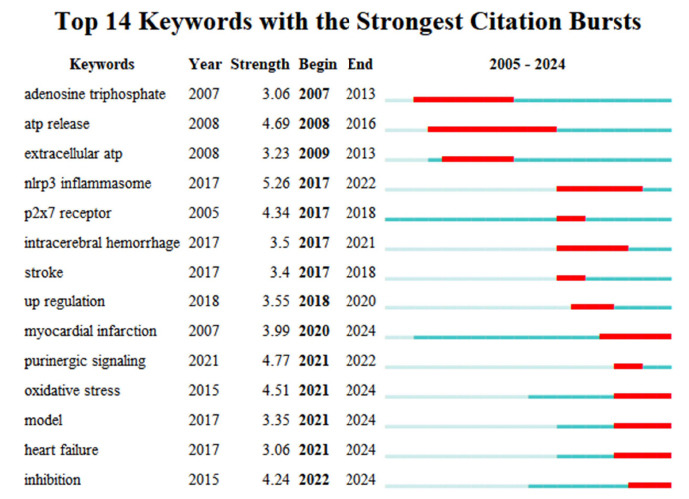
Keywords with the strongest citation bursts.

**Table 1:** Information on the top 10 journals.

**Rank**	**Publication Titles**	**Record Count**	**Category**	**IF/JCR**
1	Purinergic Signaling	31(8.36%)	Neuroscience, Cell Biology	3.0/Q2
2	International Journal of Molecular Sciences	15(4.04%)	Chemistry, Molecular Biology	4.9/Q2
3	Frontiers in Physiology	9(2.43%)	Medicine, Physiology	3.2/Q3
4	Journal of Neuroinflammation	7(1.89%)	Neuroscience, Neurology	9.3/Q1
5	American Journal of Physiology-renal Physiology	6(1.62)	Medicine, Physiology	3.7/Q2
6	PLoS One	6(1.62)	Multidisciplinary, Multidisciplinary	2.9/Q3
7	Frontiers in Pharmacology	5(1.35%)	Medicine, Pharmacology (medical)	4.4/Q2
8	American Journal of Physiology-heart and Circulatory Physiology	5(1.35%)	Medicine, Cardiology and Cardiovascular Medicine	4.1/Q2
9	Faseb Journal	5(1.35%)	Biochemistry, Genetics and Molecular Biology, Molecular Biology	4.4/Q2
10	Journal of Immunology	5(1.35%)	Medicine, Immunology	3.6/Q2

**Note:** All impact factors are the latest impact factors released by Clarivate in 2023.

**Table 2:** Top 10 keywords in terms of publications.

**Rank**	**Keyword**	**Count**
1	P2X7 receptor	154
2	Activation	94
3	ATP	93
4	Inflammation	66
5	Expression	61
6	Extracellular ATP	50
7	Nlrp3 inflammasome	40
8	Injury	33
9	Hypertension	32
10	Receptor	32

## LIST OF ABBREVIATIONS

AF: Atrial Fibrillation
AMI: Acute Myocardial Infarction
APCs: Antigen-presenting Cells
BBG: Brilliant Blue G
CNS: Central Nervous System
CVDs: Cardiovascular Diseases
DRG: Dorsal Root Ganglion
EMMPRIN: Extracellular Matrix Metalloproteinase Inducer
GDP: Gross Domestic Product
MAPK: Mitogen-activated Protein Kinase
MMP: Matrix Metalloproteinase
NLRP3: NOD-like Receptor Protein 3
P2X7R: P2X7 Receptor
PASMCs: Pulmonary Artery Smooth Muscle Cells
ROS: Reactive Oxygen Species
SCG: Superior Cervical Ganglia
siRNA: Small Interfering RNA
WoSCC: Web of Science Core Collection
